# The association of genetic polymorphisms in interleukin-1 receptors type 1 and type 2 with age-related hearing impairment in a Taiwanese population: a case control study

**DOI:** 10.1186/s40463-020-00410-z

**Published:** 2020-04-06

**Authors:** Ning-Chia Chang, Hua-Ling Yang, Chia-Yen Dai, Wen-Yi Lin, Hsun-Mo Wang, Chen-Yu Chien, Kuen-Yao Ho

**Affiliations:** 1 Department of Otorhinolaryngology, School of Medicine, College of Medicine, Kaohsiung Medical University, Kaohsiung, Taiwan; 2 Department of Otorhinolaryngology, Kaohsiung Municipal Siaogang Hospital, Kaohsiung, Taiwan; 3 Health Management Center, Kaohsiung Municipal Siaogang Hospital, Kaohsiung, Taiwan; 4 Division of Hepatobiliary and Pancreatic Medicine, Department of Internal Medicine, Kaohsiung Medical University Hospital, Kaohsiung, Taiwan; 5 Department of Internal Medicine, School of Medicine, College of Medicine, Kaohsiung Medical University, Kaohsiung, Taiwan; 6 Health Management Center, Kaohsiung Medical University Hospital, Kaohsiung, Taiwan; 7 Department of Otorhinolaryngology, Kaohsiung Medical University Hospital, No. 100, Tzyou 1st Road, Kaohsiung, 807 Taiwan

**Keywords:** Age-related hearing impairment, Interleukin-1 receptor, Single nucleotide polymorphism

## Abstract

**Background:**

Age-related hearing impairment (ARHI) is a major disability among the elder population. Chronic inflammation is an important factor in the development of ARHI. Interleukin-1 (IL-1) plays a key role in inflammation and may be associated with ARHI. The aim of this study is to analyze the associations of single nucleotide polymorphisms (SNPs) of IL-1 receptor genes with ARHI in an elderly population in Taiwan.

**Method:**

Participants ≥65 years of age were recruited for audiometric tests and genetic analyses. The bilateral pure-tone average (PTA) of high-tone hearing levels was calculated for ARHI evaluation. The associations of SNPs of the IL-1 receptor type 1 gene (IL1R1) (rs3917225 and rs2234650) and type 2 gene (IL1R2) (rs4141134 and rs2071008) with ARHI were analyzed in 182 ARHI-susceptible (case) and 176 ARHI-resistant (control) participants.

**Results:**

The G allele of IL1R1 rs3917225 showed a decreased risk of ARHI after adjustments for sex, age, and noise exposure. The GG genotype of IL1R1 rs3917225 in all hereditary models and the TT genotype of IL1R2 rs2071008 in the recessive model also showed decreased risks of ARHI after adjustments.

**Conclusion:**

These findings suggest that IL1R1 and IL1R2 polymorphisms may contribute to the decreased risk of ARHI in the elderly population.

## Introduction

Age-related hearing impairment (ARHI), or presbycusis, is one of the major disabilities among the elderly population. The prevalence of hearing impairment has been reported to be 34% in people 65 years of age or older, and it increases to 72% in people 85 years of age or older [1, 2]. In Taiwan, the prevalence of hearing impairment is reportedly as high as 78% among the population 65 years of age or older [3]. Understanding the mechanisms of ARHI may help in the prevention and treatment of ARHI, hence decreasing its prevalence.

The detailed mechanisms of ARHI development remain unclear. Noise, toxic substances, hypoxia, and chronic inflammation may increase inner ear oxidative stress, produce reactive oxygen species (ROS), lead to the necrosis and apoptosis of inner ear cells, and result in presbycusis [4–6]. Experimental inner ear inflammation studies have shown the in vivo production of tumor necrosis factor-α (TNF-α), interleukin-1 β (IL-1β), and IL-6 in the cochlea, along with synergic leukocyte infiltration [7]. Interleukin-1 (IL-1) plays a key role in inflammation and autoimmune diseases by activating the expression of genes associated with the innate and adaptive immune response [8]. The IL-1 superfamily comprises the agonist IL-1α and IL-1β and their antagonist IL-1Ra. Both IL-1 agonists can bind to IL-1 receptor type 1 and the “decoy” receptor type 2 [9]. Some authors found that the transplantation of the thymus into mice may alter the expression of IL-1 receptor type 2 on CD4+ T cells and recover the hearing in mice with ARHI [10]. Accordingly, alternations in the expression of IL-1 receptor type 1 and type 2 may be associated with the development of ARHI.

The polymorphisms of the IL-1 receptor type 1 gene (IL1R1) and type 2 gene (IL1R2) have been reported to be associated with various diseases, such as knee osteoarthritis (OA), osteonecrosis of the femoral head, and IgA nephropathy [11–13].

In single nucleotide polymorphism (SNP) studies, the allele analysis and genotype analysis were performed. The allele analysis compared the frequency of the minor allele between the case and control groups. The genotype analysis investigated the association between the SNPs and target disease using multiple genetic models. The genetic models may include different inheritance patterns, such as codominant, dominant, and recessive. For example, in the knee OA study, the allele analysis demonstrated that the minor G allele of SNP IL1R1 rs3917225 had a higher frequency in the knee OA patients than in the controls (41.3% versus 33.7%) and showed an increased risk of knee OA. And the minor G allele of IL1R1 rs3917225 was linked to an increased risk of knee OA based on the results of the dominant model [12].

However, no study on the association of IL1R1 and IL1R2 genetic polymorphisms with ARHI has been reported to date. We hypothesized that the genetic polymorphisms of interleukin-1 receptors may affect the individual’s susceptibility to ARHI. The purpose of the present study was to investigate the association of IL1R1 and IL1R2 SNPs with ARHI in an elderly population in Taiwan.

## Material and methods

### Subjects

The subjects of this study were recruited from the clients who received national annual health examinations performed by the health management center in a metropolitan hospital. The national annual health examinations were free of charge for subjects older than 65 years of age, with funding provided by the Health Promotion Administration, Ministry of Health and Welfare, Taiwan. We recruited volunteers who were 65 years of age or older and who agreed to participate in the study to receive additional pure-tone audiometric tests and fill out self-reported questionnaires about their medical history of ear diseases and history of noise exposures. Participants with histories of otologic diseases were excluded from this study later in the process of data analysis. Participants with dementia and those who could not sit independently to receive the audiometric tests were also excluded from this study.

### Audiometric assessments

Pure-tone audiometry was performed in sound-attenuating booths by trained medical technicians using standard procedures that met the requirements of the Council of Labor Affairs, Executive Yuan, Taiwan. The audiometric data were recorded at frequencies of 500, 1000, 2000, 3000, 4000, 6000, and 8000 Hz. The bilateral high-frequency (4000, 6000, and 8000 Hz) pure-tone average (PTA) was calculated. The participants were then divided into three groups according to the average high-frequency hearing levels. Participants in the worst quartile of hearing level were classified into the age-related hearing impairment group (ARHI group), while those in the best quartile of hearing level were classified into the ARHI-resistant group. The others were classified into the intermediate group. The ARHI and ARHI-resistant groups were chosen for case-control analysis.

### Genotyping

IL1R1 and IL1R2 genetic polymorphisms rs3917225, rs2234650, rs4141134 and rs2071008 were selected as the target SNPs, which were referenced from an earlier study [14]. Blood samples were obtained from all the participants with written consent. Each specimen was collected in an ethylenediaminetetraacetic acid (EDTA) tube and centrifuged (2000 g, 20 min). The buffy coat was isolated, and DNA was extracted using a commercial DNA extraction kit (Gentra Corp., Minneapolis, Minn, USA). Genotypes for the selected polymorphisms were screened with the ABI TaqMan SNP genotyping assays (Applied Biosystems, Foster City, Calif., USA). The extracted DNA and genotyping assays were added to TaqMan universal PCR master mix (Roche, Branchburg, N.J., USA) according to the manufacturer’s instructions. The genotyping procedures were then performed by using the ABI PRISM 7500 real-time PCR system (Applied Biosystems). The results were analyzed using ABI 7500 System sequence detection software version 1.2.3 (Applied Biosystems).

### Statistical analysis

All data were input to a computer and analyzed using the IBM® SPSS® statistics software package version 20.0 (International Business Machines Corp., Armonk, N.Y., USA). Continuous data were analyzed using the independent-sample Student’s t-tests. Categorical data were computed using the two-sided χ2 test. Genetic analyses were performed using PLINK software [15]. The calculation of odds ratios (ORs) and 95% confidence intervals (95% CI) and the adjustments for potentially confounding factors were performed with logistic regressions. The level of statistical significance was set at *p* < 0.05.

## Results

A total of 713 volunteers, including 400 men (56.1%) and 313 women (43.9%), were included in the present study. The mean age of the participants was 72.04 ± 5.96 years (range 65–97 years). The average high-tone hearing level was 62.36 ± 19.92 dB (range 16.67–111.67 dBHL). Among the participants, 232 (32.5%) people claimed to have been exposed to occupational noises over 85 dBSPL before retirement. In the participants who were exposed to occupational noises, 178 were men (44.5% of the male participants; 76.7% of total noise-exposed participants), and 54 were women (17.3% of the female participants; 23.3% of total noise-exposed participants). Other demographic data are presented in Table 1.

**Table 1:** Demographics of the participants

	Male	Female	Total
Number	400 (56.1%)	313 (43.9%)	713 (100%)
Age (Years)	72.42 ± 6.14	71.56 ± 5.71	72.04 ± 5.96
Bilateral PTA average (dBHL)	67.96 ± 18.87	55.27 ± 18.93	62.36 ± 19.92
Occupational noise exposure	178 (44.5%)	54 (17.3%)	232 (32.5%)
ARHI grouping			
ARHI susceptible	142 (35.5%)	40 (12.8%)	182 (25.5%)
Intermediate	197 (49.2%)	158 (50.5%)	355 (49.8%)
ARHI resistant	61 (15.2%)	115 (36.7%)	176 (24.7%)
Genotype			
IL1R1			
rs3917225			
AA	130 (32.5%)	112 (35.8%)	242 (33.9%)
AG	188 (47.0%)	137 (43.8%)	325 (45.6%)
GG	60 (15.0%)	46 (14.7%)	106 (14.9%)
Undetermined	22 (5.5%)	18 (5.8%)	40 (5.6%)
rs2234650			
CC	225 (56.2%)	146 (46.6%)	371 (52.0%)
CT	141 (35.2%)	136 (43.5%)	277 (38.8%)
TT	25 (6.2%)	20 (6.4%)	45 (6.3%)
Undetermined	9 (2.8%)	11 (3.5%)	20 (2.8%)
IL1R2			
rs4141134			
TT	356 (89.0%)	277 (88.5%)	633 (88.8%)
TC	32 (8.0%)	21 (6.7%)	53 (7.4%)
CC	0 (0)	0 (0)	0 (0)
Undetermined	12 (3.0%)	15 (4.8%)	27 (3.8%)
rs2071008			
GG	223 (55.8%)	188 (60.1%)	411 (57.6%)
GT	138 (34.5%)	99 (31.6%)	237 (33.2%)
TT	16 (4.0%)	13 (4.2%)	29 (4.1%)
Undetermined	23 (5.8%)	13 (4.2%)	36 (5.0%)
Allele			
IL1R1			
rs3917225			
A	448 (59.3%)	361 (61.2%)	809 (60.1%)
G	308 (40.7%)	229 (38.8%)	537 (39.9%)
rs2234650			
C	591 (75.6%)	428 (70.9%)	1019 (73.5%)
T	191 (24.4%)	176 (29.1%)	367 (26.5%)
IL1R2			
rs4141134			
T	744 (95.9%)	575 (96.5%)	1319 (96.1%)
C	32 (4.1%)	21 (3.5%)	53 (3.9%)
rs2071008			
G	584 (77.5%)	475 (79.2%)	1059 (78.2%)
T	170 (22.5%)	125 (20.8%)	295 (21.8%)

### Case-control study

There were 182 (25.5%) participants classified into the ARHI-susceptible group as cases (mean age = 75.34 ± 6.48 years; average PTA = 88.19 ± 10.39 dBHL; male/female = 142 (78.0%)/40 (22.0%)), and 176 (24.7%) participants with the best hearing, classified into the ARHI-resistant group as the controls (mean age = 68.92 ± 4.21 years; average PTA = 37.44 ± 8.01 dBHL; male/female = 61 (34.7%)/115 (65.3%)). The participants in the control group were significantly younger than those in the case group (t test, *p* < 0.001). The sex structure was quite different between the case and control groups, in which women had better hearing than men (chi-square; *p* < 0.001). The distribution of noise exposure history (Y/N) was also different between these two groups. There were more participants with noise exposure history in ARHI group (38.5%) than those in the control group (22.2%) (chi-square; *p* = 0.001).

### Genetic analyses

#### Allele analysis

Our analyses showed no significant association of the alleles of SNPs rs2234650, rs4141134 or rs2071008 with ARHI susceptibility in the present study, regardless of the adjustments for sex, age and noise exposure. Although there was no significant association of rs3917225 with ARHI susceptibility in the crude analysis, it revealed a significant association between rs3917225 and ARHI susceptibility after the adjustments. Participants with the G allele of rs3917225 had a decreased risk of ARHI (adjusted OR = 0.587; 95% CI = 0.383–0.901; *p* = 0.015). The results of allele analyses are presented in Table 2. The genetic distribution in the present study followed Hardy-Weinberg equilibrium.

**Table 2:** Relationships between alleles of IL-1 receptor genetic polymorphisms and age-related hearing impairment

Gene	SNPs	Allele	ARHI	Control	Crude		Adjusted^a^	
					OR (95% CI)	P	OR (95% CI)	p
IL1R1	rs3917225	A	216 (63.2%)	186 (57.1%)	1.000		1.000	
		G	126 (36.8%)	140 ((42.9%)	0.775 (0.568–1.057)	0.107	0.587 (0.383–0.901)	0.015*
	rs2234650	C	270 (75.4%)	225 (71.7%)	1.000		1.000	
		T	88 (24.6%)	89 (28.3%)	0.934 (0.664–1.313)	0.694	1.028 (0.654–1.616)	0.905
IL1R2	rs4141134	T	343 (96.3%)	333 (96.2%)	1.000		1.000	
		C	13 (3.7%)	13 (3.8%)	0.971 (0.444–2.125)	0.941	0.595 (0.209–1.699)	0.332
	rs2071008	G	276 (78.4%)	261 (79.6%)	1.000		1.000	
		T	76 (21.6%)	67 (20.4%)	1.073 (0.741–1.552)	0.710	1.062 (0.641–1.760)	0.816

a: Logistic regression; adjusted for age, sex and noise exposure

*: *p* < 0.05

#### Genotype analysis

Multiple genetic models, including codominant, dominant, and recessive inheritance patterns, were used in the genotype analysis. The analyses of genotypes showed no significant difference in the target SNPs between groups in the crude analysis. However, after adjusting for sex, age and noise exposure, rs3917225 and rs2071008 showed significant associations with ARHI. The participants with the homozygous GG genotype at rs3917225 had a decreased risk of ARHI, regardless of the inheritance model, after the adjustments. At rs2071008, the TT genotype was associated with a lower risk of ARHI in the recessive inherence model after the adjustments (adjusted OR = 0.187; 95% CI = 0.037–0.944; *p* = 0.042). There was no significant association under any inheritance model of SNP rs2234650 or rs4141134 with ARHI susceptibility, regardless of the adjustments for sex, age and noise exposure. The results of the genotype analyses are presented in Table 3.

**Table 3:** Relationships between genotypes of IL-1 receptor genetic polymorphisms and age-related hearing impairment

Gene	SNP	Model	Genotype	ARHI	Control	Crude		Adjusted^a^	
						OR (95% CI)	p	OR (95% CI)	p
IL1R1	rs3917225	Codominant	AA	66 (38.6%)	53 (32.5%)	1.000		1.000	
			AG	84 (49.1%)	80 (49.1%)	0.843 (0.525–1.354)	0.480	0.637 (0.329–1.232)	0.180
			**GG**	**21 (12.3%)**	**30 (18.4%)**	**0.562 (0.289–1.093)**	**0.089**	**0.374 (0.146–0.956)**	0.040*
		Dominant	AA	66 (38.6%)	53 (32.5%)	1.000		1.000	
			**AG + GG**	**105 (61.4%)**	**110 (67.5%)**	**0.767 (0.489–1.202)**	**0.246**	**0.546 (0.301–0.991)**	0.047*
		Recessive	AA+AG	150 (87.7%)	133 (81.6%)	1.000		1.000	
			**GG**	**21 (12.3%)**	**30 (18.4%)**	**0.621 (0.339–1.136)**	**0.122**	**0.420 (0.182–1.966)**	0.041*
	rs2234650	Codominant	CC	100 (55.9%)	91 (52.9%)	1.000		1.000	
			CT	70 (39.1%)	73 (42.4%)	0.882 (0.565–1.378)	0.583	0.982 (0.542–1.780)	0.952
			TT	9 (5.0%)	8 (4.7%)	0.511 (0.149–1.758)	0.287	0.526 (0.127–2.171)	0.374
		Dominant	CC	100 (55.9%)	91 (52.9%)	1.000		1.000	
			CT + TT	79 (44.1%)	81 (47.1%)	0.888 (0.583–1.351)	0.578	1.012 (0.582–1.759)	0.966
		Recessive	CC + CT	170 (95.0%)	164 (95.3%)	1.000		1.000	
			TT	9 (5.0%)	8 (4.7%)	1.085 (0.409–2.881)	0.869	1.098 (0.348–3.463)	0.873
IL1R2	rs4141134	Codominant	TT	165 (92.7%)	160 (92.5%)	1.000		1.000	
			TC	13 (7.3%)	13 (7.5%)	0.940 (0.408–2.165)	0.885	0.500 (0.163–1.535)	0.226
			CC	0 (0)	0 (0)	NA	NA	NA	NA
		Dominant	TT	165 (92.7%)	160 (92.5%)	1.000		1.000	
			TC + CC	13 (7.3%)	13 (7.5%)	0.940 (0.408–2.165)	0.885	0.500 (0.163–1.535)	0.226
		Recessive	TT + TC	178 (100%)	173 (100%)	1.000		1.000	
			CC	0 (0)	0 (0)	NA	NA	NA	NA
	rs2071008	Codominant	GG	104 (59.1%)	104 (63.4%)	1.000		1.000	
			GT	68 (38.6%)	53 (32.3%)	1.224 (0.768–1.950))	0.395	1.416 (0.769–2.609)	0.264
			TT	4 (2.3%)	7 (4.3%)	0.479 (0.120–1.907)	0.297	0.190 (0.032–1.110)	0.065
		Dominant	GG	104 (59.1%)	104 (63.4%)	1.000		1.000	
			GT + TT	72 (40.9%)	60 (36.6%)	1.200 (0.775–1.858)	0.414	1.294 (0.736–2.227)	0.370
		Recessive	GG + GT	172 (97.7%)	157 (95.7%)	1.000		1.000	
			**TT**	**4 (2.3%)**	**7 (4.3%)**	**0.522 (0.150–1.816)**	**0.306**	**0.187 (0.037–0.944)**	0.042*

a: Logistic regression; adjusted for age, sex and noise exposure

*: *p* < 0.05

## Discussion

ARHI is a multifactorial condition representing the end result of multiple intrinsic (e.g., genetic predisposition) and extrinsic factors (e.g., noise exposure) acting on the inner ear and leading to the accumulation of damages in the pathway of auditory signal transduction [6]. Chronic inflammation, hypoxia, noise, and toxic substances may increase oxidative stress in the inner ear, cause the production of reactive oxygen species, lead to necrosis and apoptosis of inner ear cells, and result in ARHI [4–6]. In the present study, we confirmed the hypothesis that the genetic polymorphisms of IL-1 receptors are associated with susceptibility to ARHI. These results suggest that the development of ARHI is related to the inflammatory pathway and is underlain by individual genetic differences.

IL-1 is a central mediator of innate immunity and inflammation. As a multifunctional proinflammatory cytokine, IL-1 plays a key role in inflammation and autoimmune diseases by activating the expression of genes associated with the innate and adaptive immune response [8, 16]. The biological activity of the cytokine IL-1 is medicated by its receptors [17]. IL-1 receptor type 1 affects NF-κB signaling by combining with IL-1 on the cell surface and upregulates inflammation [18]. The IL1R1 and IL1R2 genes encode cytokine receptors for IL-1. A number of studies have been performed to examine the role of the SNP rs3917225:A > G in the IL1R1 gene. Nakki et al. [19] showed that the G allele is associated with a decreased risk of severe hand osteoarthritis. Na et al. [12] found that the G allele increases the risk of knee osteoarthritis. In our study, the homozygous variant GG genotype of rs3917225 was associated with a decreased risk of ARHI, but the AG genotype showed an insignificant association with ARHI. The possible explanation might be that the GG genotype of rs3917225 decreased the risk of ARHI in the recessive pattern, and the effect of GG genotype overcame the effect of the AG genotype, resulted in the significance in the dominant model. It is not clear how this SNP affects the development of ARHI. Vasilyev et al. [14] analyzed the relationship between IL1R1 genetic polymorphisms and the expression level of membrane-bound receptors and found that rs3917225 did not show any association with the expression level of IL1R1. Dinarello et al. [20] found that IL1R1 was not abundantly expressed on the cell surface. These findings suggest that rs3917225 does not change the quantity of IL1R1, but it may alter some quality of the receptor instead. We postulate that this genetic variant might affect the affinity of IL-1 to IL-1 receptor 1, hence blocking the upregulation of inflammation.

Interleukin-1 receptor 2 is a molecular decoy that traps IL-1β and does not initiate subsequent signaling events, thereby suppressing the inflammatory response [11, 13]. Many studies have reported the associations of IL1R2 with certain diseases. Xie et al. [13] reported that the GA genotype of rs4851527:A > G exhibited increased IgA nephropathy (IgAN) risk, whereas the GA genotype of rs3218977:G > A showed decreased IgAN risk. Another study reported that the IL1R2 SNP rs11674595:T > C showed an increased risk of osteonecrosis of the femoral head (ONFH) [11]. It was reported that the minor allele T of rs2071008:T > G decreased the risk of coronary heart disease in younger adults (≤55 years of age) in the study by Chen et al. [21]. In our study, we found that rs2071008 decreased the risk of ARHI in a recessive hereditary pattern. The finding that the TT genotype of rs2071008 decreased ARHI risk in the present study was similar to the result from the study mentioned above. In the study of Vasilyev et al., homozygotes for the major allele of rs2071008 showed a lower level of membrane-bound IL1R2 on CD14^+^ monocytes in lipopolysaccharide-stimulated PBMC cultures [14]. From previous findings, we postulate that the individuals with the TT genotype of rs2071008 may have a higher level of membrane-bound IL1R2 for IL-1 binding, hence enhancing the effect of inflammation suppression.

The association of SNPs with the disease may be organ specific. In an Iranian study, the authors reported that some haplotypes of SNPs IL-1A rs1800587, IL-1B rs1143634 and IL1R1 rs2234650 were associated with systemic sclerosis [22]. Another study found that the haplotype of three IL1R2 SNPs rs4141134, rs11674595, and rs7570441 was associated with an increased risk on depressive symptom trajectories in oncology patients and family caregivers [23]. These reports indicated that IL1R1 rs2234650 might be associated with muscular diseases while IL1R2 rs4141134 might be relating to psychologic diseases. However, no association of IL1R1 rs2234650 and IL1R2 rs4141134 with ARHI was found in the present study.

Several limitations existed in the present study. First, the sample size of our study was relatively small (182 cases and 176 controls). Second, although the history of noise exposure was adjusted during static analyses, the detailed time and dose of noise exposed could not be ascertained. More male participants had occupational noise exposure and hearing loss than females. The interaction of sex and noise exposure on ARHI might be another confounding factor that should be taken into consideration. The effects of age, sex, and noise exposure might be too enormous that masks the effect of SNPs on ARHI. Therefore, we adjusted for age, sex, and noise exposure history simultaneously in the regression analysis. We believed that the results from the adjusted analysis may be closer to the true associations of SNPs with ARHI. Another limitation was that other potential risk factors of hearing loss (e.g., the habit of smoking) could not be integrated and analyzed in the present study.

In summary, we found that SNPs rs3917225 of IL1R1 and rs2071008 of IL1R2 were associated with a decreased risk of ARHI. The effect of ARHI protection might be via inflammation suppression by either decreasing the affinity of IL-1 to the IL1R1 receptor or increasing the presence of membrane-bound IL1R2 for IL-1 to bind. In a phase I/II clinical trial, the authors reported that anakinra (an IL-1 receptor antagonist) therapy in corticosteroid-resistant autoimmune inner ear disease patients was effective in a small cohort of patients and that plasma IL-1β level was associated with both clinical hearing response and disease relapse [24]. Anti-inflammatory agents such as IL-1 receptor antagonists may have potential in the prevention and/or treatment of ARHI and are worth further study.

## Conclusion

In the present study, we found that the genetic polymorphisms of IL1R1 and IL1R2 were associated with ARHI in an elderly population in Taiwan. The minor G allele and GG genotype of SNP IL1R1 rs3917225 and the homozygous variant TT genotype of IL1R2 rs2071008 in recessive inheritance pattern demonstrated decreased risks of ARHI. In contrast, SNPs rs2234650 of IL1R1 and rs4141134 of IL1R2 showed no association with ARHI.

## Data Availability

Not applicable.
